# Evaluation of Olive Oil-Based Formulations Loaded with Baricitinib for Topical Treatment of Alopecia Areata

**DOI:** 10.3390/pharmaceutics17040475

**Published:** 2025-04-05

**Authors:** Negar Beirampour, Mireia Mallandrich, Paola Bustos-Salgado, Valeri Domínguez-Villegas, Núria Garrós, Roya Mohammadi-Meyabadi, Beatriz Clares-Naveros, Maria Nuria Romero-Olid, Francisco J. Pérez-Cano, Marina Girbal, Maria José Rodríguez-Lagunas, Joaquim Suñer-Carbó, Ana Cristina Calpena

**Affiliations:** 1 Departament de Farmàcia i Tecnologia Farmacèutica, i Fisicoquímica, Facultat de Farmàcia i Ciències de l’Alimentació, Universitat de Barcelona, Av. Joan XXIII 27-31, 08028 Barcelona, Spain; nbeirabe7@alumnes.ub.edu (N.B.); mireia.mallandrich@ub.edu (M.M.); paola_bustos_salgado@ub.edu (P.B.-S.); nuria.garros@ub.edu (N.G.); rmohammo31@alumnes.ub.edu (R.M.-M.); jsuner@ub.edu (J.S.-C.); 2 Institut de Nanociència i Nanotecnologia, Universitat de Barcelona (UB), 08028 Barcelona, Spain; beatrizclares@ugr.es; 3 Facultad de Ciencias Químicas e Ingeniería, Universidad Autónoma del Estado de Morelos, Av. Universidad 1001, Cuernavaca 62209, Mexico; valeri.dominguez@uaem.mx; 4 Departamento Farmacia y Tecnología Farmacéutica, Facultad de Farmacia, Universidad de Granada, 18071 Granada, Spain; 5 Departamento de Estomatología, Facultad de Odontología, Universidad de Granada, Colegio Máximo, Campus Universitario de Cartuja s/n, 18071 Granada, Spain; nromero@ugr.es; 6 Departament de Bioquímica i Fisiologia, Facultat de Farmàcia i Ciències de l’Alimentació, Universitat de Barcelona (UB), 08028 Barcelona, Spain; franciscoperez@ub.edu (F.J.P.-C.); marinagirbal@ub.edu (M.G.); mjrodriguez@ub.edu (M.J.R.-L.)

**Keywords:** baricitinib, ex vivo permeation, alopecia areata, hair loss, skin irritation, in vitro Release, olive oil, hydroxytyrosol, squalene

## Abstract

**Background:** Alopecia areata is an autoimmune disorder that causes hair loss in clumps about the size and shape of a quarter. The estimated prevalence of the disorder is approximately 1 in 1000 people, with a lifetime risk of approximately 2 percent. One of the systemic therapies for alopecia areata consists of the use of glucocorticoids or immunosuppressants. **Methods:** Baricitinib (BCT) is a Janus kinase (JAK) 1 and 2 selective inhibitor used as an immunosuppressant drug. In this study, three olive oil BCT formulations (Oil A, Oil B, and Oil C, which differ in their content in squalene, tocopherol, tyrosol, and hydroxytyrosol) have been developed for topical delivery. The formulations were physicochemically characterized and the in vitro drug release and ex vivo permeation through human skin tissues were assessed. **Results:** The results showed nearly identical viscosity across all three formulations, exhibiting Newtonian behavior. The mathematical modeling used to describe the drug release profiles was the one-site binding hyperbola for all formulations. Oil-based formulations showed a slow BCT penetration into human skin. Skin integrity remained intact during the experiments, with no signs of irritation or alterations observed. In addition, all the formulations proved their efficacy in vivo. **Conclusions:** Among the formulations, Oil A demonstrated the highest ability retention capacity (*Q*_r_ = 1875 ± 124.32 ng/cm^2^) in the skin, making it an excellent candidate for further investigation in the treatment of alopecia areata.

## 1. Introduction

Alopecia areata is a common non-scarring hair disorder, regularly presenting with patches of hair loss on the scalp. It can evolve into severe forms, such as alopecia totalis (loss of all scalp hair) and alopecia universalis (loss of all scalp and body hair) [1]. Many known and unknown factors govern the development of this dermatologic condition. Environmental factors, viral infections, trauma, and genetic predisposition are all believed to contribute to its pathogenesis [2]. Current treatments options, such as broad-acting corticosteroids, are effective in mild cases. However, more severe forms of alopecia areata remain challenging to manage clinically. Recent research has focused on targeting the reversible Janus-associated kinase (JAK) pathway as a potential therapeutic approach for alopecia [3].

In alopecia areata, hair loss occurs when the immune system fails to protect hair follicles, leading to the infiltration of natural killer group 2D (NKG2D) positive CD8^+^ T cells and CD4^+^ T cells [4]. These T cells release interferon-γ, which activates Janus kinase-1 and 2 (JAK1 and JAK2) in the epithelial cells of hair follicle. This activation triggers the secretion of interleukin-15, further stimulating JAK1 and JAK3 in the T cells. IL-15 promotes the survival, proliferation, and activation of cytotoxic CD8^+^ T cells, which are key players in the autoimmune attack on hair follicles in AA [5]. Ultimately, this leads to the damage of hair follicles and subsequent hair loss [2,6]. In small clinical studies, JAK inhibitors have demonstrated potential in the treatment of alopecia [7].

Baricitinib (BCT) (2-(3-(4-(7*H*-pyrrolo[2,3-*d*]pyrimidin-4-yl)-1*H*-pyrazol-1-yl)-1-(ethylsulfonyl) zetidine-3-yl) acetonitrile), with a molecular weight of 371.42 g/mol (C_16_H_17_N_7_O_2_S), is a small molecule able to inhibit the signaling pathway of JAK [8]. BCT is an oral medication that selectively inhibits JAK1 and JAK2 and is approved for the treatment of moderately to severely active rheumatoid arthritis in adults in over 70 countries [9]. Furthermore, in the European Union and Japan, it has been approved for treating moderate-to-severe atopic dermatitis in adults [10]. Moreover, BCT has shown anti-inflammatory properties in patients with autoimmune diseases [11,12]. It has been demonstrated that JAKs have a critical function in cytokine signaling. Consequently, inhibiting JAKs would lead to the blocking of specific cytokines and thereby reducing the inflammation [13]. Jabbari A et al. revealed a very successful clinical response to the treatment of alopecia areata with BCT [14]. BCT is rapidly absorbed after oral administration and reaches peak plasma concentrations at approximately 1 h. BCT oral bioavailability is 79% and it is moderately bound (about 50%) to plasma proteins, mainly albumin [15]. Olive oil is rich in fatty acids, such as its oleic, stearic, bioactive and antioxidant components. It has been used for hundreds of years to treat various pathologies such as inflammation, hypertension and gout and topical conditions such as wounds, sores, and infections on the mucosa or skin [15,16]. In cosmetics, it is used for its moisturizing properties to help strengthen the hair and increase its elasticity, helping prevent breakage or split ends [17,18]. Also, oleuropein present in olive oil impacts the hair-growth cycle in mice [18]. Due to its acidic components and permeability potential, olive oil might be a good carrier for treating topical conditions. Specifically, oils containing BCT seem to be a good approach to treating alopecia areata.

Lately, a few delivery systems have been investigated for topical baricitinib. Garrós et al. investigated liposomal formulations of baricitinib for ophthalmic use in Sjögren’s syndrome [19]. In another work, Mohammadi et al. developed a lipid-based solution of baricitinib (BCT-OS) for the topical treatment of psoriasis. The researchers formulated baricitinib in excipients like Transcutol^®^ P, Labrafac^®^ Lipophile WL 1349, and Lauroglycol^®^ 90 [20]. Nene et al. examined a baricitinib-loaded nanoemulgel for atopic dermatitis, with promising results in reducing inflammation [21].

These studies demonstrated the potential of topical baricitinib. However, none of them explored olive oil-based formulations, not to mention that the researchers addressed different therapeutic targets. The novelty of our work lies in its unique approach of using olive oil-based formulations of baricitinib for the topical treatment of alopecia areata, addressing the specific limitations of the existing research. Therefore, the main purpose of this work was to study the effectiveness of three olive oil-based formulations in combination with BCT and Transcutol^®^ P to stimulate hair growth as part of the treatment for alopecia areata. Olive oil with a higher content of squalene (Oil A), another containing hydroxytyrosol as major component (Oil B), and common extra virgin olive oil (Oil C) were used as part of the essayed formulations. The detailed physicochemical oil characterization, the in vitro release, the ex vivo permeation studies through skin tissues, and the histological evaluation of the effect of these oils were conducted as part of the more specific goals. In addition, we explored the antioxidant potential of the oils by DPPH analysis.

## 2. Materials and Methods

### 2.1. Chemicals and Reagents

Baricitinib (BCT) and Formic acid ammonium salt were bought at Sigma-Aldrich (Madrid, Spain). Transcutol^®^ P [Diethylene glycol monoethyl ether] was supplied by Gattefossé (Barcelona, Spain). DPPH (2,2-diphenyl-2-picrylhydrazyl hydrate) was acquired from (Sigma-Aldrich Chemie, Steinheim, Germany). Double distilled water was obtained from a Milli-Q^®^ purification system lab supply (Millipore Corporation, Burlington, MA, USA).

### 2.2. Biological Tissue for Ex Vivo Permeation Study

Human skin tissues were obtained from the Barcelona SCIAS Hospital (Barcelona, Spain), having come from abdominal region plastic surgery on healthy women with the approval of the Bioethics Committee of the Barcelona-SCIAS Hospital (N°002; dated 17 January 2020). The skin was stored at −20 °C until the experiments were carried out.

### 2.3. Olive Oils

The three oils used in this study were kindly donated by Isanatur (Navarra, Spain). Oil A and B are extracts of organic olive pulp and skin, rich in squalene, tocopherol, hydroxytyrosol, and tyrosol (Table 1), with Oil A having a higher content of squalene and Oil B a higher content of hydroxytyrosol and tyrosol. Oil C is a common extra virgin olive oil.

### 2.4. Preparation of Olive Oil-Based Formulations

**Figure 1:**
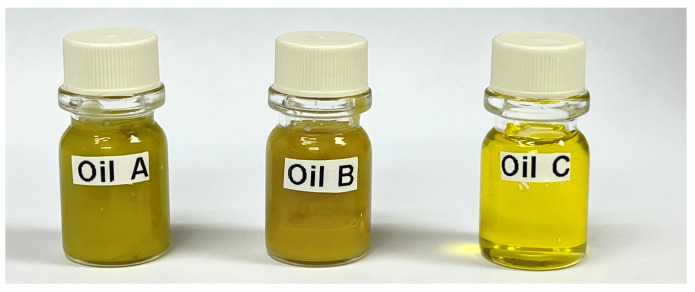
Visual presentation of the three elaborate formulations.

The oils formulations were prepared by dissolving 10 mg of baricitinib (BCT) with 1 mL of organic solvent (Transcutol^®^ P) and then mixed with 4 mL of the corresponding olive oil (Figure 1). Then, the solutions were sonicated in an Elma Transonic Digital S T490 DH ultrasonic bath (Elma, Singen, Germany). Finally, the oils were filtered through syringe filters with a 0.45 μm pore size (Millex^®^, Sigma-Aldrich, Madrid, Spain).

### 2.5. Physicochemical Characterization of Oils Formulations

#### 2.5.1. Chromatographic Operating Conditions for the Quantification of BCT

The content of BCT in the formulations was assessed by chromatographic analysis. BCT was extracted from 0.5 mL of the formulations with 2 mL of Transcutol^®^ P, then the mixture was vortexed for 2 min and analyzed by High Pressure Liquid Chromatography (HPLC) (Waters Alliance 2695, Waters Corporation, Milford, MA, USA) with a fluorescence detector (Jasco FP-1520, JASCO Corporation, Hachioji, Tokyo, Japan) at an excitation wavelength of 310 nm and an emission wavelength of 390 nm [22]. The mobile phase consisted of Ammonium Formate 10 mM pH 7 and Acetonitrile (75:25 *v*/*v*) under isocratic elution at a flow rate of 1 mL/min in a Symmetry C18 column (4.6 mm ID × 75 mm length, 3.5 μm particle size). The injection volume was 10 μL.

#### 2.5.2. pH Measurements

To assess the stability of oil formulations the pH was measured in triplicate, at room temperature, using a digital pH-meter Crison micro pH 2001 (Crison Instruments SA, Alella, Spain). The measurements of the formulations were conducted via direct immersion of the device electrode in samples at 25 ± 0.1 °C. The determinations were carried out with the formulations freshly prepared and after 60 days of storage at 4 ± 1 °C. Results were expressed as the mean ± standard deviation (SD) of the three replicates. Any changes in pH data can demonstrate some instability over the storage period.

#### 2.5.3. Extensibility Test

The extensibility study was carried out separately for the three formulations intended for the topical application. To evaluate this property, a volume of 30 μL of an oil sample was placed between two glass plates, while focusing as much as possible and avoiding the sliding of the plates. Then, the force generated on the upper plate was measured by adding known weights (10, 20, 50, 100 g). The weights were removed after 60 s, and the diameter was registered. The increase in spreading area was plotted as a function of the increasing weights applied. Different mathematical functions were evaluated using GraphPad Prism^®^ software version 6.0 (GraphPad Software Inc., San Diego, CA, USA), and the one with the highest R^2^ value was the following:

(1) $$y=\frac{B_{max}\times x}{K_{d}+x}$$

in which *B_max_* is the maximal surface and *d* is the weight required to reach the half-maximal surface. Each sample was measured at room temperature in triplicate with each weight [23].

#### 2.5.4. Rheological Studies

Rheological measurements were performed using a Haake Rheostress^®^ 1 rheometer (Thermo Fisher Scientific, Karlsruhe, Germany) linked to a thermostatic circulator Thermo Haake Phoenix II + Haake C25P and a computer PC with Haake Rheowin^®^ Data Manager v. 4.87 software (Thermo Fisher Scientific, Karlsruhe, Germany). Rotational tests were carried out to determine the viscosity and flow behavior of the formulations. Steady-state measurements were addressed with a cone-and-plate geometry set up (C60/2°Ti: 60 mm diameter, 2° angle). Each sample was placed between the cone-and-plate sensor system (0.105 mm gap) for 5 min. Steady-state viscosity (η, mPa·s) was determined from the constant shear section at 100 s^−1^. The shear stress (τ) was measured as a function of the shear rate (γ). Viscosity curves (η = f(γ)) and flow curves (τ = f(γ)) were recorded at 25 ± 0.1 °C. The shear rate ramp program included a 3 min ramp-up period from 0 to 100 s^−1^, 1 min constant shear rate period at 100 s^−1^, and 3 min ramp-down from 100 to 0 s^−1^.

### 2.6. Antioxidant Properties

The 2,2-Diphenyl-1-picrylhydrazyl (DPPH) assay was used to measure the antioxidant activity of BCT, and the oils with or without BCT (placebo oils). BCT was dissolved in ethanol to obtain various concentrations (10, 100 and 1000 μM) while formulations Oil A, Oil B, and Oil C, and the placebo formulations (respective oils without BCT, Oil A^−^, B^−^, and C^−^) were diluted with ethanol (10, 100 and 1000 ppm). Then, 500 μL of diluted samples and 1500 μL of DPPH ethanolic solution were incubated in 96-well microliter plates at 37 °C for 30 min. When DPPH reacts with an antioxidant compound, which can donate hydrogen, it is reduced. The changes in color (from a deep violet to a light yellow) were measured at 517 nm on a UV/visible light spectrophotometer (Spectronic Genesys 8, Thermo Fisher Scientific, Rochester, NY, USA). Results were expressed as percentage of Radical Scavenging Activity (*RSA*), calculated as follows:

(2) $$RSA\%=\frac{A_{0}-A_{s}}{A_{0}}\times 100$$

where *A*_0_ is the absorbance of the control and *A_s_* is the absorbance of the samples at 517 nm [24,25]. The experiment was performed in triplicate.

### 2.7. In Vitro Release Studies of Oils Fomulations

The release study of the formulations of oils was investigated employing vertical Franz cells with a diffusional area of 0.64 cm^2^ (Franz Diffusion Cells 400; Crown Glass, Somerville, NJ, USA) and a dialysis membrane (12 kDa, Dialysis Tubing Visking, Medicell International Ltd., London, UK). Prior to the test, the membranes were hydrated for over 24 h in a mixture of Transcutol^®^ P and phosphate-buffered solution (PBS) pH 7.4 (1:1 *v*/*v*) was employed to fill the receptor compartment, which was constantly stirred (700 r.p.m.). Subsequently, 0.3 mL of the oils were inserted in the donor compartment; the system was kept at 32 ± 5 °C. At predefined time intervals up to 52 h, aliquots of 0.3 μL were taken from the receptor compartment and replaced with the same volume of Transcutol^®^ P:PBS 7.4. The amount of BCT released from the different oils in each sample was quantified by HPLC analytical method described in Section 2.5.1. The mean ± SD of five replicates was used to calculate the results. To assess the release kinetics, the data were fitted to the mathematical models: Zero order, and Boltzmann.

(3) Y=Yintercept+(Slope·X)

(4) $$Y=Bottom+\frac{\left(Top-Bottom\right)}{1+e^{\left(\frac{V50-X}{Slope}\right)}}$$

where Y is amount of BCT released at time (*t*), X is the time. *Bottom* is the lowest amount of drug released and top is the maximum amount of BCT released; *V*50 is the time at which the drug is halfway released.

Some amodelistic parameters were also calculated: mean BCT release time from each oil (MRT), the area under the curve from release time, 0, to 52 h (AUC 0–52), the efficiency (E), which was calculated as the area under the curve of the release profile between the period of 0–52 h, with regard to the total amount of drug released within the same period of time, expressed as the percentage (Equation (5)).

(5) $$E=\frac{{AUC}_{0}^{52}}{A_{52}\times T_{52}}$$

where *E* is the drug release efficiency, *AUC* is the area under the curve within the interval 0–52 h, *A*_52_ is the amount release at 52 h, and *T*_52_ is the time 52 h.

Analysis of variance and Tukey’s *t*-test were calculated to evaluate the significant differences between release profiles of all the formulations. Data were considered statistically significant at *p* < 0.05.

### 2.8. Ex Vivo Permeation of Oils Formulation Through Skin

Ex vivo studies were performed using human skin stored at −20 °C prior to cutting it to 400 μm thickness with a dermatome. The experiments were conducted in vertical Franz diffusion cells with a diffusional surface area of 0.64 cm^2^ for 24 h at 32 ± 0.5 °C under continuous stirring, in accordance with sink conditions. Skin tissues were positioned as the membrane between the two compartments of a Franz cell, with the epidermis side in contact with the donor chamber, covered with a laboratory film to prevent evaporation. Same as in vitro release studies, Transcutol^®^ P:PBS 7.4 (1:1) was used to fill the receptor compartment. A total of 500 μL of the test formulations was applied once the temperature of the skin surface had equilibrated to 32 ± 0.5 °C. At each sampling interval of up to 28 h, a volume of 300 μL of the receptor medium was withdrawn and an equal volume of fresh receptor medium was added. The amount of BCT permeated and retained in the skin was determined by Fluorimetry method (see Section 2.5.1). Results are reported as mean and standard deviation of six replicates (*n* = 6) in the donor compartment [26].

#### 2.8.1. Calculation of the Permeation Parameters

The cumulative amounts of BCT from the permeation profile model under infinite dose conditions were analyzed using the GraphPad Prism^®^ software version 9.5.1 (GraphPad Software Inc., San Diego, CA, USA). The permeation profiles were structured by plotting the cumulative amount of BCT permeated (μg) versus time (h); from this, the flux was calculated as the slope of the linear part by linear regression and the lag time (*T_l_*, h), which was calculated as the x-intercept of the linear regression. Other permeation parameters, such as permeability coefficient (*K_p_*, cm/h), partition, and diffusion parameters (*P*_1_ and *P*_2_), were calculated according to the following equations:

(6) $$K_{p}=\frac{J}{C_{0}}$$

(7) $$P_{2}=\frac{1}{6\cdot Tl}$$

(8) $$P_{1}=\frac{J}{A\times C_{0}\times P_{2}}$$

where *J* (μg/h) is the flux across the skin membrane, *A* is the exposed tissue surface area (cm^2^), *C*_0_ (μg/mL) is the initial concentration of the formulation tested in the donor compartment. It was assumed that in the infinite dose approach, the drug concentration in the receiver compartment was negligible compared to that in the donor compartment. The predicted steady-state plasma concentration (*C_ss_*, μg/mL) for each formulation was estimated in accordance with Equation (9) [27].

(9) $$C_{ss}=\frac{J\times TSA}{{Cl}_{p}\times A}$$

where *J* is the flux, *TSA* is a hypothetical area of application, *Cl_p_* is the plasma clearance of BCT reported in human and *A* is the diffusional area of the Franz cells.

#### 2.8.2. Amount of BCT Retained in the Skin

At the end of the permeation study, the skin tissues were carefully removed from the Franz cells to determine the amount of BCT retained in the skin. To this end, the skin surface was cleaned three times with a gauze soaked in a 0.05% solution of sodium lauryl sulfate and distilled water. Excess skin around the diffusion area was removed, and the diffusion area was gently blotted with a filter paper to provide a dried surface area prior to weighting. BCT retained in the skin was extracted with Transcutol^®^ P under sonication for 15 min using an ultrasonic water bath, after the entire skin surface was pricked with a needle. The resulting solutions were analyzed by HPLC with a fluorescence detector (see Section 2.5.1), and the amount of BCT retained in the skin, *Q*_r_ (μg/cm^2^) was determined according to Equation (10) [28]:

(10) $$Q_{r}=\frac{\frac{EX}{PX}}{A}\times \frac{100}{R}$$

where the ratio *EX*/*PX* is the extracted amount of the drug expressed in μg; *A* is the diffusion area in cm^2^; and *R* is the recovery rate of BCT in the skin [8].

### 2.9. Tolerance and Efficacy Studies

#### 2.9.1. In Vivo Tolerance Study by Evaluating the Biomechanical Properties of Human Skin

The tolerance study was conducted to analyze the effect of the excipients of the three formulations oils, without BCT, on the skin. The test was conducted on female volunteers with healthy skin. The study was approved by the Ethics Committee of the University of Barcelona (IRB00003099) in accordance with the recommendations of the Declaration of Helsinki, and all volunteers provided signed written informed consent forms [29]. Trans epidermal water loss (TEWL, g/m^2^/h) and stratum corneum hydration (SCH, arbitrary units, AU) were the parameters measured on the flexor side of the left forearm and at 5, 15, 30, 60, 90, and 120 min after the application of the oil formulations. Measurements were carried out with a Tewameter (TEWL-DermaLab module) (Cortex Technology, Hadsund, Denmark) and a Corneometer CM 825 (Courage-Khazaka Electronic GmbH, Cologne, Germany). Baseline readings were recorded before application of the oils. Results are reported as mean ± SD (*n* = 6).

#### 2.9.2. Efficacy Study of Oils Formulations on Hair Growth in Mice

For the assessment of the efficacy of BCT oil formulations, DQ8-Dd-villin-IL-15tg mice were employed, which were kindly provided by Dr. Valerie Abadie at The University of Chicago (Chicago, IL, USA). These mice expressed higher levels of IL-15; although it is a mice model of celiac disease, some of these mice presented alopecia throughout their life [30] and have been used to mimic alopecia areata. This study was approved by the CEEA-UB (Ref. 186/20). Two male and female mice were randomly distributed into 5 groups: placebo group presenting alopecia (REF), Oil A, Oil B, Oil C, or placebo. All groups were treated topically once a day for 15 days with 100 μL of the formulations (PBS, Oil A, Oil B, Oil C, or a mixture of the oils without BCT, respectively) to the dorsal region using a calibrated micropipette. Photographic documentation was performed on Day 1 (baseline) and Day 15 (endpoint), and tissue sampling was performed at the last day of the study. At the day of sacrifice, animals were anesthetized intramuscularly with ketamine (90 mg/kg) (Merial Laboratories SA, Barcelona, Spain) and xylazine (10 mg/kg) (Bayer AG, Leverkusen, Germany), their blood was collected by cardiac puncture for hematological analysis, and the back skin was removed for histological evaluation.

#### 2.9.3. Histological Analysis

Skin samples underwent standardized histological processing protocols, including fixation in 10% neutral buffered formalin for 24 h, sequential dehydration through ascending ethanol gradients, xylene clearing, and paraffin embedding. Tissue sections (5 μm thickness) were obtained using a calibrated rotary microtome and stained with hematoxylin and eosin (H&E). The number of hair follicles containing a clearly identifiable hair shaft within the follicular canal, and follicular structures without a visible hair shaft but maintaining an intact follicular architecture, named empty follicles, were evaluated using a light microscope Olympus BX41 equipped with an Olympus XC50 camera (Olympus Co., Tokyo, Japan), following the description denoted by the authors of [31].

### 2.10. Statistical Analysis

Experimental data were analyzed using GraphPad Prism software, version 9.5.1. One-way ANOVA fallowed Tukey’s multiple comparison test was used to compare mean values across the three treated groups. A *p*-value of <0.05 was considered statistically significant.

## 3. Results

### 3.1. Physicochemical Characterization of Oils

The olive oil-based formulations were analyzed for drug content by HPLC. As can be seen in Table 2, Oil A contained more BCT than the other oils. Despite the preparation methodology being consistent across all formulations, differences in the BCT content among the formulations were observed. This can be attributed to variations in the olive oil compositions that might influence the solubility and distribution of BCT in the oil. We conducted Bartlett’s statistical test to assess the homogeneity of variances among the different formulations. The results indicated no significant statistical differences in BCT content, suggesting that the observed variations are within the expected range of experimental variability. Furthermore, Tukey’s Multiple Comparison Test was also conducted, and revealed significant differences between formulations A and C, while no significant differences were observed between A and B or B and C. This supports the hypothesis that intrinsic variability in oils composition and minor procedural differences can lead to variations in BCT content

The pH values of Oil A, Oil B, and Oil C were 6.42 ± 0.10, 5.5 ± 0.07, and 6.25 ± 0.15, respectively. And the pH remained constant during the two-month storage period at 4 °C.

**Figure 2:**
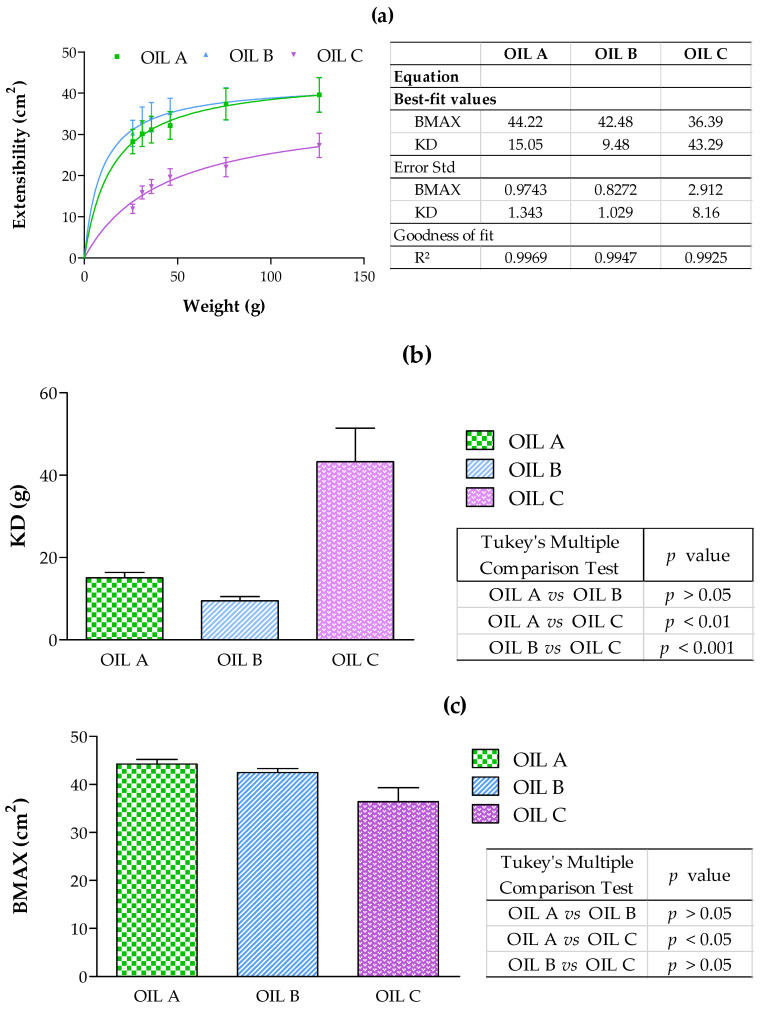
(**a**) Extended area in cm^2^ after applying 26, 31, 36, 46, 76, and 126 g of weight (*n* = 3, mean ± SD). (**b**) Statistical analysis expressed by KD = weights of Oil A, B, C, and (**c**) statistical analysis expressed by BMAX = maximal surface of oils formulation. Statistically significant differences were *p* < 0.05. One-way ANOVA = analysis of variance test, followed by Tukey’s multiple comparison test are expressed in the figures to show the statistical differences between them.

All the oil formulations were in accordance with the one-site binding (hyperbola) model. Figure 2 shows the extensibility results. No significant statistical differences were observed between Oil A and Oil B, and both of them were more extensible than Oil C.

#### Rheological Behavior

**Figure 3:**
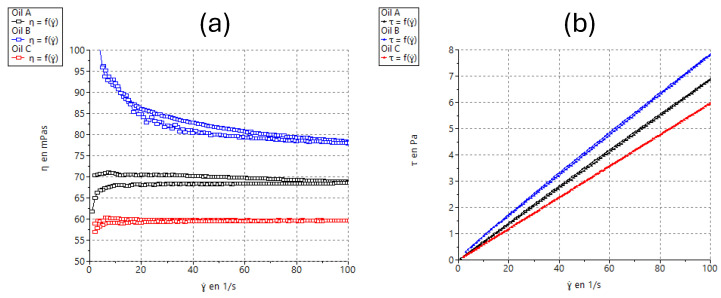
Rheograms of tested formulations: (**a**) viscosity curve of Oil A, B, and C; (**b**) flow curves of Oil A, B, and C.

Viscosity measurements are used to determine whether the material exhibits linear (Newtonian) viscous behavior (shear stress profile *n* = 1) or whether it exhibits (*n* + 1) power law behavior [32]. All three formulations demonstrated Newtonian flow behavior. Concerning viscosity measurements, the nature of the oil is important in the final properties of semisolid formulations. It is believed that the viscosity of the oil phase has a dominant effect on the physicochemical characteristics of semisolid formulations. The potential dependence of the oil viscosities on the shear rate is shown in Figure 3 and viscosity values at 100 s^−1^ in Table 3.

### 3.2. Antioxidant Test

In the antioxidant test, it was observed that the oil formulations A, B, and C, containing Transcutol^®^ P and BCT, presented higher DPPH free radical reduction values than those exhibited by the placebo formulation (Oil A^−^, B^−^ and C^−^) at the highest concentration tested, 1000 ppm (Table 4). It should be noted that the antioxidant values of BCT alone, in ethanolic solution, did not reflect an antioxidant property. Nevertheless, the presence of BCT in the oil formulation potentiated the antioxidant activity of Oils A and C. Oil B presented the highest antioxidant activity in DPPH free radical trapping capacity at a concentration of 1000 ppm with or without BCT. A statistical analysis using Tukey’s multiple comparison test to evaluate the antioxidant activity of the oil formulations (Tables S1–S3). When analyzing the placebos, Oils A and B exhibited notable antioxidant activity, whereas Oil C showed only a slight effect. When baricitinib was incorporated into the oils, the antioxidant activity increased for A and C formulations. Although Oil B demonstrated a slightly higher capacity, no significant differences were observed between Oils A and B. However, both Oils A and B were significantly different from Oil C. When comparing the baricitinib-containing oils to their respective placebos, we found that the incorporation of baricitinib enhanced the antioxidant activity in Oils A and C. In contrast, no significant differences were observed for Oil B when compared to its placebo.

### 3.3. In Vitro Release of Oils Formulations

**Figure 4:**
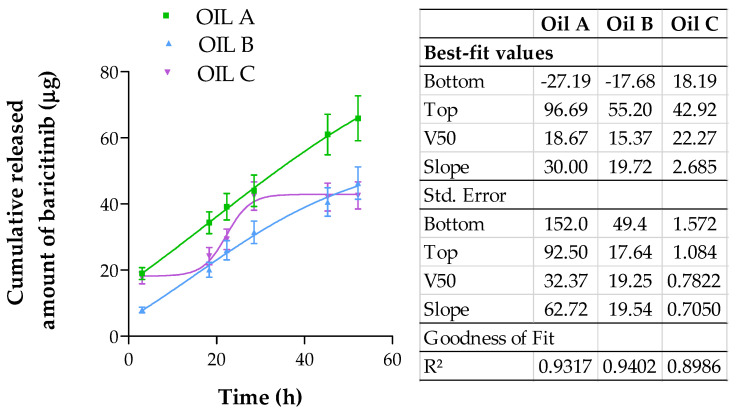
Release profiles of BCT from Oils A, B and C: cumulative released amount of BCT (μg) vs. time (h). Results are expressed as mean ± SD (*n* = 5).

Figure 4 shows the release profile of BCT from the different oil formulations assayed. The results were fitted to different mathematical models and the best one was chosen based on the R^2^ value. The results of the model fitting showed that all formulations were adjusted to the Boltzmann sigmoidal fitting model.

In this study, the formulations showed a sustained release of BCT over the 52 h period. The duration of the in vitro drug release test should be long enough to characterize the release profile. To facilitate a comparison of the biopharmaceutical characteristics of each formulation, various parameters for adjustment (as detailed in Table 5) were computed, including the mean release time (MRT), area under the curve (AUC), and efficacy (E). Oil A had the lowest MRT value, 19.30 h, compared to 30.80 and 26.60 h for Oil B and Oil C, respectively. This indicates that Oil A is capable of releasing the drug at a faster rate. This formulation also resulted in better bioavailability in the tissue (Table 6). Oil A exhibited a remarkably higher AUC value than Oils B and C, having a value of 2158.0 μg/h, Oil B a value of 1399.3 μg/h, and Oil C a value of 1675 μg/h. Efficiency results revealed that Oil A had the highest value, at 62.90%, and was the oil with the fastest release and the one that released the most amount of drug. Oils B and C, even with the differences in the process, in the end released the same amount of final drug. Table 5 also shows the different model-independent parameters for the three oils, the result being that Oil A was statistically different from Oil B and Oil C for all amodelistic parameters.

### 3.4. Ex Vivo Permeation of Oils Through Human Skin

**Figure 5:**
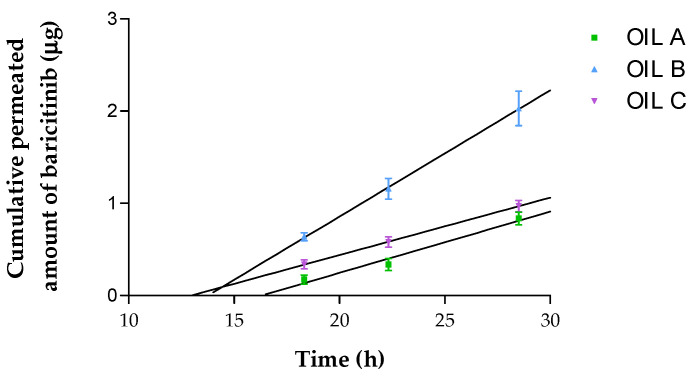
Permeation profiles of BCT from Oil A, B, and C; cumulative permeated amount of BCT (μg) vs. time (h). Results are expressed as mean ± SD (*n* = 6).

The permeation profile of the three oils is depicted in Figure 5. The different permeation parameters were calculated and the statistically significant differences are reported in Table 6. Oil B is the one with the highest flux (*J*), permeability coefficient (*K_p_*) and theoretical plasma predicted concentration in human steady state (*C_ss_*). Oil A achieved the highest amount of BCT retained in the human skin at 28 h (*Q_ret_*), followed by Oil B and Oil C; all of the oils showed significant differences between them. Similarly, the *K_p_* was statistically different between all oils tested. In contrast, only the *J* and *C_ss_* from Oil A and Oil C had no statistical differences between them (*p* > 0.05).

### 3.5. Skin Tolerance in Humans and Hair Growth Efficacy in Mice of Oil-Based Formulations

#### 3.5.1. Human Skin Tolerance Test

**Figure 6:**
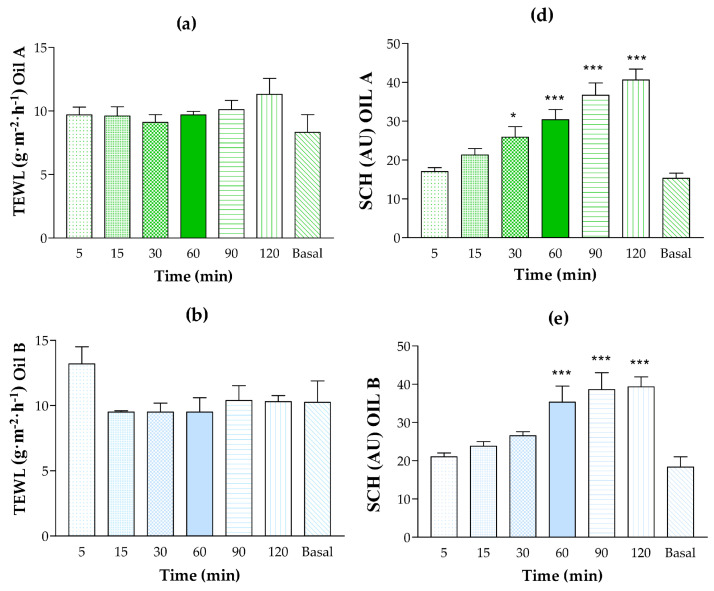

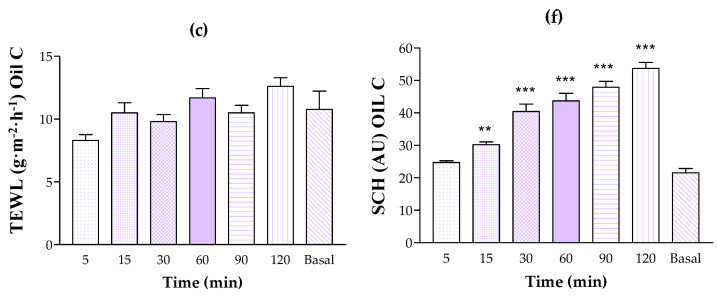
Human skin tolerance test. The evolution of biomechanical parameters was monitored before the application of the formulations and 2 h after application. Transepidermal water loss (TEWL) is expressed as g/m^2^/h. (**a**) TEWL values of Oil A. (**b**) TEWL values of Oil B. (**c**) TEWL values of Oil C. (**d**) Stratum corneum hydration (SCH) values of Oil A. (**e**) SCH values of Oil B. (**f**) SCH values of Oil C. Statistically significant differences: * *p* < 0.05, ** *p* < 0.01, and *** *p* < 0.001 versus basal value.

The tolerability of the placebo formulations was evaluated by monitoring the potential changes in the biomechanical parameters—specifically, transepidermal water loss (TEWL) and stratum corneum hydration (SCH)—before application (baseline) and at 5, 15, 30, 60, 90, and 120 min post-application of the tested formulations. This monitoring period allowed for an assessment of the initial tolerability of the vehicles. As illustrated in Figure 6, all tested oils exhibited a moisturizing effect on the skin. Regarding TEWL, which is indicative of skin barrier function [33,34,35], Oil A showed a slight increase compared to the baseline values. However, no statistically significant differences were observed, and TEWL remained below 15 g/m^2^/h, indicating that the formulation did not compromise the skin barrier. Similar results were obtained for Oil B. Oil C showed a slight tendency to increase TEWL values relative to baseline, but, as with Oils A and B, the changes were not statistically significant, and TEWL remained below 15 g/m^2^/h during the assessment period. Furthermore, none of the volunteers reported burning, itching, or visible irritation following the application of the oil formulations (A, B, C). Collectively, these findings suggest that the tested oil formulations were well tolerated. Given these promising results, future clinical studies should be conducted to confirm these preliminary findings.

#### 3.5.2. Efficacy Study of Oils Formulations on Hair Growth in Mice

**Figure 7:**
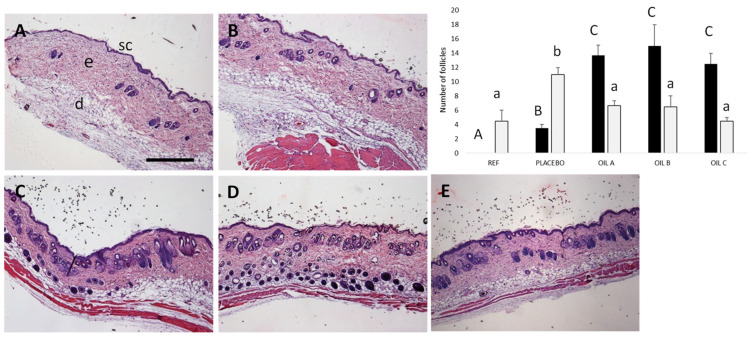
Representative histological sections of alopecic patches in the back skin of mice treated for 15 days with PBS, reference (REF) (**A**), a mixture of oils without baricitinib; placebo (**B**); Oil A (**C**); Oil B (**D**); or Oil C (**E**). Number of hair follicles with visible hair shafts and number of empty follicles are shown in gray and black bars, respectively. Different letters denote statistical differences. Skin structures: sc, stratum corneum; e, epidermis; d, dermis;. Scale bar = 100 μm, original magnification, ×40.

Finally, the oils were tested for 15 days in mice that presented hair loss in the back skin. In Figure 7, representative sections stained with hematoxylin and eosin can be observed. By day 15, the control group (PBS) exhibited significant follicular degradation, whereas treatment with Oil A demonstrated optimal histological improvements, showing enhanced follicular structure preservation and hair shaft formation. Oil B, containing higher levels of tyrosol and hydroxytyrosol, also showed follicular regeneration, and Oil C also increased the number of visible hair shafts. Moreover, mice presented clear evidence of new hair shaft formation (Figure S1), with respect to the follicular degradation and absence of hair observed in PBS controls. Placebo animals exhibited variable responses with limited structural improvements, validating the therapeutic efficacy of baricitinib-containing formulations.

## 4. Discussion

This work uniquely focuses on alopecia areata, whereas the previous studies target different conditions: Sjögren’s syndrome [19], psoriasis [20], and atopic dermatitis [21]. This specific focus allows for a tailored approach. Olive oils have been used to treat skin conditions for hundreds of years [15,16,17,18]. We developed three formulations based on two extracts of organic olive pulp and skin, rich in squalene, tocopherol, hydroxytyrosol, and tyrosol, alongside a standard extra virgin olive oil allowing for an analysis of how these components influence the formulation’s efficacy. Each of the oils contained 20% Transcutol^®^ P and 10 mg of BCT, designed for topical application in the treatment of alopecia areata to promote hair growth. Transcutol^®^ P was selected for its dual benefits: enhancing the solubility of BCT and serving as a permeation enhancer due to its proven biocompatibility [36,37]. The pH levels of the formulations were maintained between 5.5 and 6.5, aligning with the optimal range for skin application [38]. Notably, olive oils with higher acidity have been shown to significantly enhance skin permeation compared to their less acidic counterparts, positioning them as superior carriers in cutaneous drug delivery systems [39]. This property is particularly advantageous for the transdermal delivery of active compounds as previously reported for Diclofenac [40]. Our permeation study corroborates this, demonstrating that the acidic nature of the olive oil extracts used in our formulations plays a critical role in improving the bioavailability of BCT through the skin.

Extensibility is crucial for skin formulations, as it ensures a uniform distribution of the active ingredient and facilitates an even application. Furthermore, products with smooth spreading characteristics enhance patient compliance by providing a more pleasant application experience [41]. All three formulations demonstrated appropriate extensibility for dermal applications, with good retention and ease of use [42]. Oil C exhibited the lowest extensibility, indicating higher application resistance, while Oils A and B showed statistically comparable extensibility patterns.

Rheological analysis indicated that all three formulations exhibited similar properties, with viscosity curves displaying linear patterns consistent with Newtonian behavior. Viscosity is a critical parameter in topical formulations, as values that are too high or too low can negatively impact patient comfort and ease of application [43]. Among the formulations, Oil B displayed the highest viscosity, followed by Oil A and Oil C, respectively.

The antioxidant properties of these formulations play a dual role: they prevent the degradation of the product and contribute positively to skin health [44]. This is particularly important, as oxidative stress can impair the skin’s barrier function and disrupt sebaceous gland activity, potentially exacerbating conditions like alopecia areata [39]. A DPPH assay was used to evaluate the antioxidant potential of the formulations and not as a direct measure of their in vitro or in vivo efficacy. The results confirmed antioxidant activity across all three formulations, with Oil B exhibiting the highest antioxidant activity, attributable to its elevated hydroxytyrosol content. Oils A and C followed in antioxidant potency. This enhanced antioxidant capacity not only supports the stability of the formulations but also provides additional protective benefits to the skin, helping to mitigate oxidative damage.

Drug release studies showed sustained BCT release over 52 h, reflected in extended Mean Residence Time (MRT) values. Oil A achieved the highest BCT release with the shortest mean release time. While all formulations followed similar release kinetics, Oil A showed statistically significant differences compared to Oils B and C. Previous research by Jayshree Mahanty et al. has identified triterpenes and phenolic compounds as natural permeation enhancers [41]. Our study prioritizes skin retention of baricitinib, aiming for a sustained local effect, as opposed to permeation. This strategy is particularly beneficial for conditions like alopecia areata, where the drug needs to act locally within the skin, on hair follicles, rather than being absorbed systemically.

In this study, transgenic mice that expressed higher levels of IL-15 have been used to prove the efficacy of the formulations on recovering the air follicles. IL-15 is a key cytokine involved in the pathogenesis of alopecia areata, playing a crucial role in amplifying the autoimmune response against hair follicles. It has been demonstrated that IFN-γ promotes IL-15 production in hair follicles through JAK1/2 signaling, while IL-15, in turn, stimulates IFN-γ production by T cells via JAK1/3 signaling. This cycle exacerbates inflammation and contributes to the loss of immune privilege in hair follicles, ultimately leading to their destruction by CD8^+^NKG2D^+^ T cells. By inhibiting JAK1/2, baricitinib disrupts this inflammatory cascade, reducing the autoimmune attack on hair follicles and potentially reversing alopecia areata [12,45]. Baricitinib has been used in randomized controlled trials conducted in adult patients with severe AA [12]; however, some events classified as mild or moderate have been reported [46]. The use of baricitinib in topical formulation would improve the side effects while reducing the severity of the disease. Animals that did not receive baricitinib treatment showed a progressive increase in the affected area, whereas those treated with the formulations demonstrated different retention capacities. In this regard, the results of the histological analysis of the alopecic back skin of the mice revealed marked differences between the treatment groups. Our results showed that in the treatment groups, the majority of quantified follicles contained visible hair shafts, whereas in the placebo and reference groups, a higher proportion of follicles appeared empty. While it is possible that some of these empty follicles could eventually produce hair, as expected, the untreated animals exhibited an increasing number of empty follicles over time, suggesting follicular degeneration. This observation aligns with the previous findings in hair cycle research, indicating that untreated follicles in certain conditions may undergo miniaturization or involution [47]. The histological analysis supported the macroscopic observations of hair growth patterns observed in the mice. Furthermore, no differences in hematological parameters were observed in those animals, suggesting no side effect. In addition, all formulations demonstrated excellent tolerability, with no adverse effects on skin structure or integrity.

Biophysical measurements confirmed that the formulations preserved skin barrier integrity, with TEWL and hydration levels remaining within normal ranges throughout the treatment period. These findings confirm the safety and suitability of the formulations as topical agents. Among them, Oil A emerged as the most promising candidate for the therapeutic application in the treatment of alopecia areata. According to Montero-Vilchez et al., healthy skin typically exhibits TEWL values below 15 g·m^−2^·h^−1^ and hydration values around 40 AU [48]. High TEWL values indicate a compromised skin barrier that is not effectively preventing water loss, which can lead to dryness, irritation, and increased vulnerability to external irritants. Our results confirmed that none of the tested formulations disrupted skin barrier function, as TEWL values consistently fell within these acceptable ranges. The fact that the formulations maintained the skin’s integrity suggests that the oil-based formulations are a good vehicle for topical delivery of baricitinib, because a compromised barrier can lead to unpredictable drug absorption and potentially adverse effects. We observed that the developed olive oil-based formulations provided moisturizing and antioxidant properties. Adequate hydration of the stratum corneum is important for maintaining skin elasticity, softness, and overall barrier function.

While topical immunotherapy using sensitizers such as diphenylcyclopropenone (DPCP) remains a common approach for extensive alopecia areata, our findings suggest that these oil-based BCT formulations could offer an alternative treatment pathway with potential immunosuppressive effects. This alternative may be particularly valuable for patients who do not respond well to traditional therapies or who experience adverse reactions. Our study suggests that this treatment approach not only helps prevent further hair loss, but may also enhance hair density, offering a dual benefit of preserving existing hair and promoting new growth. Future research should focus on long-term efficacy and patient outcomes to better understand the full therapeutic potential of these formulations. Investigating their performance in larger, more diverse patient populations, and in combination with other therapeutic agents, could provide further insights into their role in managing alopecia areata.

## 5. Conclusions

This study focused on the development of BCT oil formulations for treating alopecia. Among the formulations, Oil A demonstrated superior performance in both drug release and skin retention, alongside notable improvements in hair growth and follicular structure. The comprehensive evaluation of its physicochemical and biological properties highlights Oil A as a promising candidate for further development. These encouraging findings support the need for continued research to advance this formulation as a potential treatment for alopecia areata.

## Figures and Tables

**Table 1:** Squalene, tocopherol, hydroxytyrosol, and tyrosol content in oils A, B, and C.

	Oil A	Oil B	Oil C
Squalene (mg/mL)	1500	1000	150
Tocopherol (mg/mL)	30	40	15
Hydroxytyrosol (mg/mL)	1	10	0.1
Tyrosol (mg/mL)	50	150	0.5

**Table 2:** Baricitinib content in Oils A, B and C.

Formulation	Baricitinib Content (μg/mL)
Oil A	219.96 ± 40.28
Oil B	158.33 ± 35.79
Oil C	149.08 ± 34.57

**Table 3:** Rheological model and viscosity of oil formulations measured at 25 °C.

Formulation	Rheological Behavior Model and Stretch Ramp-Down	Viscosity at 100 s^−1^ (mPa·s)
Oil A	Newtonian (Newton, r = 0.9999)	68.67 ± 0.10
Oil B	Newtonian (Newton, r = 0.9999)	78.15 ± 0.01
Oil C	Newtonian (Newton, r = 1)	59.64 ± 0.05

**Table 4:** Antioxidant activity of the formulations Oil A, B, and C; their placebos Oil A^−^, B^−^ and C^−^; and an ethanolic solution of baricitinib, expressed as the mean and standard deviation (SD) of DPPH% reduction in three replicates.

Sample	DPPH % of Reduction (Mean ± SD)		
	10 ppm	100 ppm	1000 ppm
Oil A^−^	−4.212 ± 1.758	−3.796 ± 0.450	10.088 ± 1.531
Oil B^−^	−5.720 ± 3.602	−0.7280 ± 0.392	17.680 ± 0.392
Oil C^−^	−1.352 ± 0.238	−4.368 ± 2.298	0.708 ± 1.261
Oil A	−8.736 ± 6.398	1.300 ± 1.250	15.184 ± 1.729
Oil B	−5.668 ± 3.235	2.808 ± 0.6619	17.836 ± 1.288
Oil C	−2.808 ± 0.2548	−0.1560 ± 1.544	12.012 ± 0.3821
BCT solution	−1.300 ± 2.944	−2.860 ± 0.9215	−13.931 ± 1.808

Oils A^−^, B^−^ and C^−^, respective placebo oils, without BCT. BTC solution = baricitinib ethanolic solution.

**Table 5:** Amodelistic parameters for Oil A, B, and C formulations and their statistical differences. Turkey’s multiples comparison test results.

Parameter	Oil A	Oil B	Oil C	Tukey’s Multiple Comparison Test		
	Mean ± SD	Mean ± SD	Mean ± SD	Oil A vs. Oil B	Oil A vs. Oil C	Oil B vs. Oil C
AUC 0–52 (μg·h)	2158.00 ± 215.4	1399.00 ± 127.30	1675.00 ± 159.30	*p* < 0.001	*p* < 0.01	*p* > 0.05
Efficiency (%)	62.90 ± 6.20	40.80 ± 4.20	48.80 ± 5.10	*p* < 0.001	*p* < 0.01	*p* > 0.05
MRT (h)	19.30 ± 1.30	30.80 ± 3.00	26.60 ± 2.70	*p* < 0.001	*p* < 0.01	*p* > 0.05
*A*_52_ (μg)	65.90 ± 6.80	46.30 ± 4.90	42.60 ± 4.10	*p* < 0.001	*p* < 0.001	*p* > 0.05

AUC 0–52 = area under the curve from time 0 to 52 h; MRT = mean release time; *A*_52_ = amount of BCT released at 52 h. Significant differences in Tukey’s multiple comparison test followed by ANOVA test analysis of variance *p* < 0.05, *p* < 0.01 and *p* < 0.001.

**Table 6:** Permeation parameters from Oils A, B, and C, expressed by their mean and standard deviation (SD).

Parameter	Oil A	Oil B	Oil C	Tukey’s Multiple Comparison Test		
	Mean ± SD	Mean ± SD	Mean ± SD	Oil A vs. Oil B	Oil A vs. Oil C	Oil B vs. Oil C
*J* (μg/h)	0.066 ± 0.011	0.137 ± 0.003	0.062 ± 0.001	*p* < 0.001	*p* > 0.05	*p* < 0.001
*J*/*sup* (μg/h/cm^2^)	0.104 ± 0.017	0.214 ± 0.005	0.097 ± 0.001	*p* < 0.001	*p* > 0.05	*p* < 0.001
*K_p_* (cm/h)	0.003 ± 0.001	0.015 ± 0.000	0.005 ± 0.000	*p* < 0.001	*p* < 0.001	*p* < 0.001
*T_l_* (h)	16.31 ± 1.65	13.76 ± 1.48	12.96 ± 1.35	*p* < 0.05	*p* < 0.01	*p* > 0.05
*P*_1_ (cm)	0.299 ± 0.005	1.232 ± 0.003	0.399 ± 0.000	*p* < 0.001	*p* < 0.001	*p* < 0.001
*P*_2_ (1/h)	0.010 ± 0.001	0.012 ± 0.001	0.013 ± 0.001	*p* > 0.05	*p* < 0.05	*p* > 0.05
*C_ss_* (ng/mL)	0.198 ± 0.033	0.409 ± 0.009	0.186 ± 0.002	*p* < 0.001	*p* > 0.05	*p* < 0.001
*Q_ret_* (ng/cm^2^)	1875.00 ± 124.32	468.75 ± 54.38	125.00 ± 12.37	*p* < 0.001	*p* < 0.001	*p* < 0.001

*Q_ret_* = the retained amount of BCT on the human skin at 28 h, *J* = Flux, *J*/*sup* = flux normalized by the diffusional area of the Franz cells, *K_p_* = permeability coefficient, *T_l_* = lag time, *P*_1_ = partition parameter, *P*_2_ = diffusion parameter, and *C_ss_* = theoretical plasma predicted concentration in human steady state. Statistically significant differences: *p* < 0.05, *p* < 0.01 and *p* < 0.001, by analysis of variance and Tukey’s multiple comparison test.

## Data Availability

The datasets generated and/or analyzed during the study are available from the corresponding author on reasonable request.

## Supplementary Materials

The following supporting information can be downloaded at: https://www.mdpi.com/article/10.3390/pharmaceutics17040475/s1, Table S1: Statistical analysis of the placebo formulations; Table S2: Statistical analysis of the oil formulations containing baricitinib; Table S3: Statistical analysis of oil formulations compared to their respective placebos; Table S4: Haematological analysis of mice treated topically with the oils for 15 days; Figure S1: Representative alopecic patches of back skin of mice before (day 1) and after the treatment (day 15) with PBS, reference (A), a mixture of oils without Baricitinib, Placebo (B), Oil A (C), Oil B (D) or Oil C (E).
