# Supplementary material for: Evaluation of Olive Oil-Based Formulations Loaded with Baricitinib for Topical Treatment of Alopecia Areata
Source: Pharmaceutics. 2025 Apr 5;17(4):475. doi: 10.3390/pharmaceutics17040475 (PMC12030606; doi:10.3390/pharmaceutics17040475)
Supplement: Supplementary file 1 [file pharmaceutics-17-00475-s001.zip › pharmaceutics-3469315-supplementary.pdf]

Supplementary Materials

Table S1. Statistical analysis of the placebo formulations.

Sample	Tukey's Multiple Comparison Test	Mean Diff.	q	P value	95% CI of diff
10 ppm	Oil A vs Oil B	1.508	1.127	P > 0.05	-4.299 to 7.315
	Oil A vs Oil C	-2.86	2.137	P > 0.05	-8.667 to 2.947
	Oil B vs Oil C	-4.368	3.264	P > 0.05	-10.18 to 1.439
100 ppm	Oil A vs Oil B	-3.068	3.877	P > 0.05	-6.502 to 0.3659
	Oil A vs Oil C	0.572	0.723	P > 0.05	-2.862 to 4.006
	Oil B vs Oil C	3.64	4.599	P < 0.05	0.2061 to 7.074
1000 ppm	Oil A vs Oil B	-7.592	11.27	P < 0.001	-10.52 to -4.668
	Oil A vs Oil C	9.38	13.92	P < 0.001	6.456 to 12.30
	Oil B vs Oil C	16.97	25.18	P < 0.001	14.05 to 19.90

Table S2. Statistical analysis of the oil formulations containing baricitinib.

Sample	Tukey's Multiple Comparison Test	Mean Diff.	q	P value	95% CI of diff
10	Oil A vs Oil B	-3.068	2.815	P > 0.05	-7.797 to 1.661
	Oil A vs Oil C	-5.928	5.44	P < 0.05	-10.66 to -1.199
	Oil B vs Oil C	-2.86	2.624	P > 0.05	-7.589 to 1.869
100	Oil A vs Oil B	-1.508	2.161	P > 0.05	-4.537 to 1.521
	Oil A vs Oil C	1.456	2.086	P > 0.05	-1.573 to 4.485
	Oil B vs Oil C	2.964	4.247	P > 0.05	-0.06452 to 5.993
1000	Oil A vs Oil B	-2.652	3.634	P > 0.05	-5.819 to 0.5149
	Oil A vs Oil C	3.172	4.346	P < 0.05	0.005096 to 6.339
	Oil B vs Oil C	5.824	7.98	P < 0.01	2.657 to 8.991

Table S3. Statistical analysis of oil formulations compared to their respective placebos.

	Oil A placebo			Oil A			Unpaired t test
**Sample**	**average**	**sd**	**n**	**average**	**sd**	**n**	**P value**
10 ppm	-4.212	1.758	3	-8.736	0.398	3	0.0122
100 ppm	-3.796	0.45	3	1.3	1.25	3	0.0027
1000 ppm	10.088	1.531	3	15.184	1.729	3	0.0187

	Oil B placebo			Oil B			
	**average**	**sd**	**n**	**average**	**sd**	**n**	**P value**
10 ppm	-5.72	3.602	3	-5.668	3.235	3	0.986
100 ppm	-0.728	0.392	3	2.808	0.662	3	0.0013
1000 ppm	17.68	0.392	3	17.836	1.288	3	0.8507

	Oil C placebo			Oil C			
	**average**	**sd**	**n**	**average**	**sd**	**n**	**P value**
10 ppm	-1.352	0.238	3	-2.808	0.255	3	0.0019
100 ppm	-4.368	2.298	3	-0.156	1.544	3	0.0579
1000 ppm	0.708	1.261	3	12.012	0.382	3	0.0001

Table S4. Haematological analysis of mice treated topically with the oils for 15 days.

	REF	Placebo	Oil A	Oil B	Oil C
**GB (cells/L)**	3.30E+09 ± 5.00E+08	4.03E+09 ± 1.33E+09	7.35E+09 ± 3.95E+09	2.65E+09 ± 5.00E+07	7.35E+09 ± 5.25E+09
**LYM (%)**	12.05 ± 0.65	20.10 ± 3.49	18.10 ± 5.80	13.70 ± 1.10	26.60 ± 4.00
**MID (%)**	37.00 ± 0.10	34.97 ± 2.87	34.90 ± 3.90	35.45 ± 5.75	32.75 ± 1.25
**GRAN (%)**	50.95 ± 0.55	44.93 ± 1.54	47.00 ± 1.90	50.85 ± 6.85	40.45 ± 2.55
**LYM (cells/L)**	3.50E+08 ± 5.00E+07	8.67E+08 ± 4.06E+08	1.55E+09 ± 1.15E+09	3.00E+08 ± 0.00E+00	2.10E+09 ± 1.70E+09
**MID (cells/L)**	7.50E+08 ± 6.50E+08	1.30E+09 ± 3.79E+08	2.40E+09 ± 1.10E+09	9.00E+08 ± 2.00E+08	2.30E+09 ± 1.60E+09
**GRAN (cells/L)**	1.75E+09 ± 2.50E+08	1.87E+09 ± 5.49E+08	3.40E+09 ± 1.70E+09	1.45E+09 ± 1.50E+08	2.95E+09 ± 1.95E+09
**GR (cells/L)**	8.01E+12 ± 3.35E+11	7.64E+12 ± 2.50E+11	7.50E+12 ± 4.85E+11	7.17E+12 ± 8.00E+10	7.42E+12 ± 1.25E+11
**HGB (g/dL)**	10.65 ± 0.25	10.33 ± 0.38	9.95 ± 0.85	9.75 ± 0.05	10.00 ± 0.10
**HCT (%)**	36.35 ± 1.85	35.13 ± 1.18	34.40 ± 2.30	33.80 ± 0.50	34.35 ± 0.25
**VCM (f/L)**	45.45 ± 0.45	46.03 ± 0.30	45.95 ± 0.15	47.20 ± 1.20	46.45 ± 0.45
**MCH (pg)**	13.25 ± 0.25	13.47 ± 0.09	13.20 ± 0.30	13.55 ± 0.25	13.40 ± 0.10
**MCHC (g/dL)**	29.30 ± 0.80	29.37 ± 0.07	28.85 ± 0.55	28.80 ± 0.30	29.05 ± 0.05
**RDW-CV (%)**	12.40 ± 0.50	12.47 ± 0.34	12.30 ± 0.60	12.00 ± 0.30	11.60 ± 0.10
**RDW-SD (fL)**	18.85 ± 0.65	19.07 ± 0.43	18.20 ± 0.00	18.85 ± 0.65	18.85 ± 0.65
**PLQ (cells/L)**	5.66E+11 ± 5.00E+09	4.98E+11 ± 2.85E+10	3.65E+11 ± 9.95E+10	6.45E+11 ± 1.51E+11	2.26E+11 ± 1.07E+11
**MPV (fL)**	6.90 ± 0.10	7.17 ± 0.15	7.35 ± 0.45	7.10 ± 0.10	7.80 ± 0.30
**PDW (fL)**	7.00 ± 0.20	7.30 ± 0.32	7.50 ± 0.00	7.15 ± 0.35	7.85 ± 0.35

**PCT (%)**	0.39 ± 0.01	0.35 ± 0.03	0.26 ± 0.06	0.46 ± 0.12	0.17 ± 0.08
**P-LCR (%)**	3.05 ± 0.85	4.03 ± 0.55	3.90 ± 1.00	4.65 ± 0.15	1.50 ± 1.50
**P-LCC (cells/L)**	1.65E+10 ± 4.50E+09	1.97E+10 ± 3.48E+09	1.25E+10 ± 5.00E+08	3.00E+10 ± 8.00E+09	9.50E+09 ± 5E+08

White blood cell count (GB); Lymphocyte (LYM); Monocyte (MID); Granulocyte (GRAN); Red blood cell count (GR); Hemoglobin concentration (HGB); Hematocrit (HCT); Mean Corpuscular Volume (MCV); Mean Corpuscular Hemoglobin (MCH); Mean Corpuscular Hemoglobin Concentration (MCHC); Red Blood Cell Distribution width Repeat Precision (RDWCV); Red Blood Cell Distribution Width STDEV (RDW-SD); Platelet count (PLQ), Mean platelet volum (MPV); Platelet distribution width (PDW); Plateletcrit (PCT); Large platelet ratio (P-LCR); Large platelet (P_LCC).

Heparin-treated blood was immediately used to count platelets and white and red blood cells and related variables using an automated hematology analyzer (Spincell3, MonLab, Barcelona), following the manufacturer's instructions.

Supplementary Figure

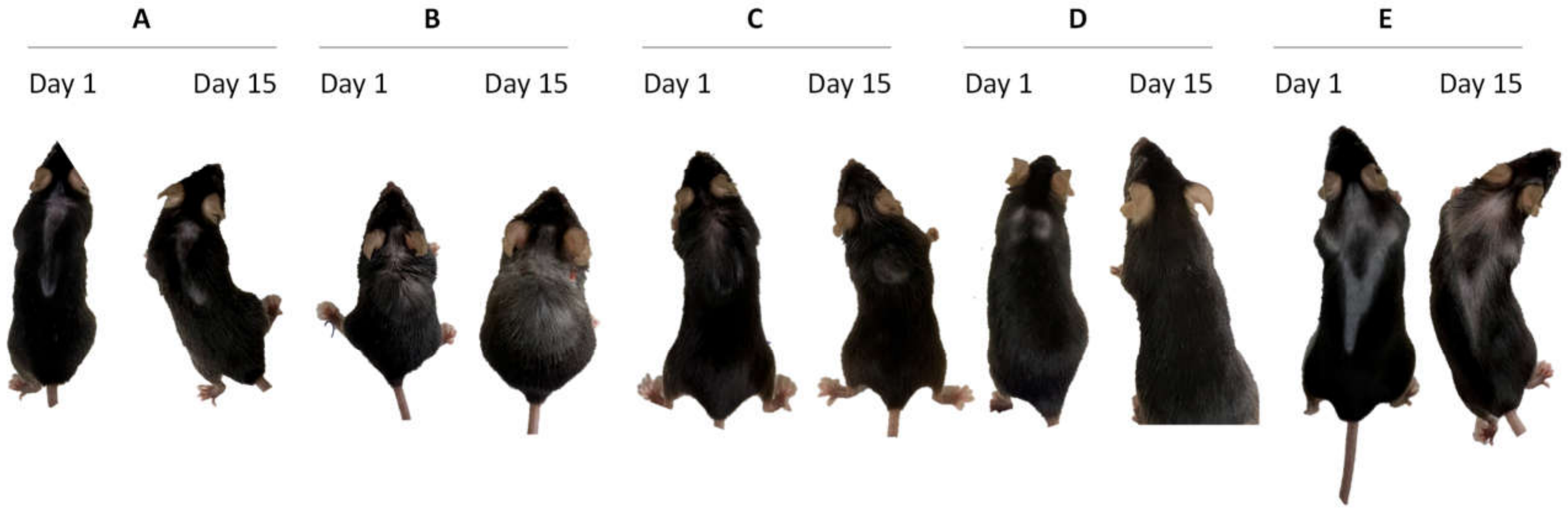

Figure S1. Representative alopecic patches of back skin of mice before (day 1) and after the treatment (day 15) with PBS, reference (**A**), a mixture of oils without Baricitinib, Placebo (**B**), Oil A (**C**), Oil B (**D**) or Oil C (**E**). º
